# A Novel Coating System Based on Layered Double Hydroxide/HQS Hierarchical Structure for Reliable Protection of Mg Alloy: Electrochemical and Computational Perspectives

**DOI:** 10.3390/ma17051176

**Published:** 2024-03-02

**Authors:** Maryam Chafiq, Aisha H. Al-Moubaraki, Abdelkarim Chaouiki, Young Gun Ko

**Affiliations:** 1 Materials Electrochemistry Laboratory, School of Materials Science and Engineering, Yeungnam University, Gyeongsan 38541, Republic of Korea; 2 Department of Chemistry, Faculty of Sciences—Alfaisaliah Campus, University of Jeddah, Jeddah 21589, Saudi Arabia

**Keywords:** organic–inorganic layer, layered double hydroxide, anticorrosion property, functional material, DFTB simulations

## Abstract

Growing research activity on layered double hydroxide (LDH)-based materials for novel applications has been increasing; however, promoting LDH layer growth and examining its morphologies without resorting to extreme pressure conditions remains a challenge. In the present study, we enhance LDH growth and morphology examination without extreme pressure conditions. By synthesizing Mg-Al LDH directly on plasma electrolytic oxidation (PEO)-treated Mg alloy surfaces and pores at ambient pressure, the direct synthesis was achieved feasibly without autoclave requirements, employing a suitable chelating agent. Additionally, enhancing corrosion resistance involved incorporating electron donor–acceptor compounds into a protective layer, with 8-Hydroxyquinoline-5-sulfonic acid (HQS) that helps in augmenting Mg alloy corrosion resistance through the combination of LDH ion-exchange ability and the organic layer. DFT simulations were used to explain the mutual interactions in the LDH system and provide a theoretical knowledge of the interfacial process at the molecular level.

## 1. Introduction

Lately, there has been a significant focus on two-dimensional (2D) materials due to their attractive physical, electrical, chemical, and optical characteristics. In recent decades, researchers have dedicated significant effort to enhancing multifunctional materials, particularly focusing on layered double hydroxides (LDHs) as a prominent example of 2D materials. LDH has garnered extensive attention in materials science research fields owing to its robust interlayer coupling and chemical stability. Exploiting these properties, LDH has emerged as an ideal candidate for serving as a physical barrier to inhibit the corrosion of metal substrates. Nevertheless, despite all these great benefits, LDH is unable to be the best solution or to broaden its scope of applicability in practical applications due to the necessity of using an autoclave, which places additional demands on the industry sectors’ environmental acceptability. Considering this particular issue, the in situ formation of Mg-Al LDH in the presence of EDTA has been demonstrated in this study, circumventing the hydrothermal autoclave conditions that present a technical barrier to the industrial application of such coating systems’ remarkable advantages. Within this framework, a significant challenge for LDH in these applications revolves around fully leveraging layered coating materials with substantial strength, serving as robust pillars in nanotechnology. Yet, achieving this without imposing restrictive conditions is paramount. Therefore, expanding the scope of coating methods to broaden their applicability stands as a central focus for advancement. This is accomplished with the aim of responding to the extreme requirements of metals and materials engineering.

Because of their outstanding strength-to-weight ratio and biocompatibility, magnesium (Mg) and its alloys are considered as highly promising metallic materials applicable in aeronautics, automotive engineering, and corrosion fields [1,2,3,4,5]. It is also applicable to the development of new energy vehicles in response to the growing need for lightweight materials and environmental conservation [6,7]. Additionally, because of their high biological compatibility and non-toxic characteristics, the utilization of magnesium alloys is extremely encouraging in the chemical and pharmaceutical industries [8,9]. The widespread applicability of Mg alloys is significantly hindered by their pronounced susceptibility to corrosion, which is primarily attributed to their low standard potential of 2.36 V when compared to a normal hydrogen electrode [10]. Their limited application options are caused by their poor corrosion resistance and significant chemical reactivity. In the past few years, numerous research groups have recommended alloying and various surface treatment methods to enhance the corrosion resistance of Mg-based materials in aggressive environments. These methods include chemical conversion coatings, sol-gel processes, organic/polymer coatings, chemical/physical vapor deposition, anodizing, and plasma electrolytic oxidation (PEO). Distinguished by their efficacy, elevated chemical stability, strong adhesion, and satisfactory mechanical properties, these coatings stand out among various anti-corrosion techniques, and so forth [11,12]. Significantly, PEO emerges as a remarkably effective technique for generating substantial oxide coatings on lightweight metals, notably acclaimed as one of the most promising surface treatments for Mg alloys. Its superiority over conventional methods lies in several aspects, including the exceptional adherence of the inorganic layer to the substrate, the simplicity of one-step processing, its relatively low cost, and the ease of control. Although there has been progress, it is still extremely difficult to achieve both a high and long-lasting inhibition, particularly given that the extraordinary performance of LDH in the case of corrosion is still in doubt due to the inherent flaws in the perpendicular nano-petal architecture, which include gaps, which make corrosion resistance, particularly over a long period of time, not really achievable. Hence, to improve the corrosion resistance provided by the inorganic layer produced on the substrate using the PEO/LDH process, it is crucial to incorporate an additional treatment to enhance the overall effectiveness of corrosion resistance.

Multifunctional hybrid materials, combining organic and inorganic elements, are recommended for their unique advantages, making them valuable in high-end applications. This uniqueness results from the fact that they blend the inorganic and organic qualities of the materials of which they are constructed and provide a unique perspective on a variety of materials. These substances cover a wide spectrum, ranging from materials with a high concentration of inorganic compounds, resembling ceramics closely, to those with a minimal presence of inorganic compounds, thus aligning more with polymers. Over the past years, there has been a rigorous examination of this category of materials. Wang et al. [13] introduced a new method involving Ca-Al LDH hybrid self-healing microcapsules, where tung oil serves as the core and ethyl cellulose as the wall, synthesized using the solvent evaporation technique. The study focused on evaluating the self-healing abilities and chlorine adsorption properties of these hybrid microcapsules within cement-based materials. The results indicated that the nano-layered Ca-Al LDH in the hybrid wall provided additional nucleation sites for cement hydration. The combination of self-healing and chlorine resistance in the hybrid microcapsules enhanced the corrosion resistance of steel bars. The exothermic process during the formation of hybrid materials exhibited excellent binding stability. Moreover, the chlorine adsorption capacity of the microcapsules was improved due to the interface charge redistribution within their hybrid materials. In addition, Zhong’s research group [14] introduced a microcapsule demonstrating exceptional efficiency in chloride responsiveness and synergistic corrosion resistance. A thorough evaluation of the synthesized material encompassed an analysis of its chemical structure, triggering performance and a corrosion resistance mechanism [15,16]. The exploration into the hybridization mechanism and corrosion resistance involved experimental characterization along with first-principles calculations, offering valuable insights into the intricate details of the developed microcapsule. Kaseem et al. [17] reported the development of flower and rod-like structures on the inorganic layer when it was immersed in solutions with varying concentrations of albumin and sodium phosphate. This immersion resulted in the formation of protective composites on the AZ31 alloy. According to research conducted by Du et al. [18], incorporating Ca^2+^ into a sodium-alginate hydrogel coating loaded with vancomycin on PEO-treated Mg alloy can lead to additional cross-linking of the coating, consequently improving corrosion resistance. Zhang et al. [19] found that the formation of the Mg-Al LDH coating on the surface of PEO-coated AZ31 alloy showed great promise in enhancing its chemical stability when exposed to a 5 wt.% NaCl solution. In the field of catalysis, scientists are actively leveraging these exceptional characteristics to devise efficient methods for the removal and treatment of organic pollutants using LDHs. Shao et al. [20] synthesized Zn-Ti LDH, achieving complete photodegradation of methylene blue within 100 min, surpassing the performance of ZnO and TiO_2_ catalysts. Asif and colleagues [21] successfully produced a composite of graphene oxide and Co-Al LDH via the co-precipitation method. The resulting catalyst displayed a degradation efficiency of approximately 71% for methylene blue after 60 min of UV irradiation exposure. However, LDHs encounter a significant challenge due to the inefficient separation of photogenerated charges, thereby limiting their photocatalytic performance [22]. In the field of corrosion, creating a crystallographic ab-oriented LDH film poses challenges because LDH crystallites usually align perpendicular to coating surfaces. This alignment leads to micro-intercrystallite defects within the LDH coating, which could permit corrosive anions to infiltrate the substrate, causing significant deterioration and compromising long-term protection. For this reason, a novel strategy for modifying LDH materials is essential to achieve superior corrosion protection for magnesium alloys. Given the context provided, it leads to speculation about the potential to enhance the electrochemical stability of the PEO/LDH-coated AZ31 Mg alloy by introducing an organic layer, exploiting the chemistry of 8-Hydroxyquinoline-5-sulfonic acid (HQS) molecules. In this work, we report the application of the fabrication of Mg-Al-LDH nano-petals employing EDTA as a chelating molecule on the PEO surface for excellent anticorrosion properties through the application of a structural design taking inspiration from the nature of the HQS compound. The study also delved into the anticorrosion mechanism of the prepared composite coating. Despite the considerable research on molecular self-assembly, there remains a gap in understanding the molecular-level details of the interfacial interaction mechanism and the influence of inter- and intra-molecular interactions on structural stabilization. In this study, various levels of theory have been performed to explore the inter--/intra-molecular interactions, charge transfer behavior, and intrinsic characteristics of the molecular network of HQS. Density functional theory (DFT) calculations are conducted to gain insights into how the geometric structure of the organic molecule influences its interaction with the inorganic layer. Experimental techniques and theoretical calculations confirmed the structure and inter-/intra- molecular interactions between HQS and the LDH layer. This robust linking system exhibited impressive effectiveness in promoting the growth of the organic layer on the LDH films.

## 2. Experimental and Simulation Aspects

### 2.1. Inorganic Layer Fabrication

This study utilized materials of extra purity grade, sourced from Sigma–Aldrich, and employed them as received, without further purification. The inorganic layer fabrication process began with treating a commercially available AZ31 Mg alloy through plasma electrolysis using an electrolyte consisting of 6 g L^−1^ potassium hydroxide (KOH) and 6 g L^−1^ tripotassium phosphate (K_3_PO_4_). Prior to PEO, samples with a dimension of 30 mm × 20 mm × 4 mm were grounded and washed ultrasonically. The plasma electrolytic oxidation treatment involved applying an AC current for 360 s, with a constant frequency and a current density of 200 mA/cm^2^.

### 2.2. HQS@LDH Fabrication

**Figure 1:**
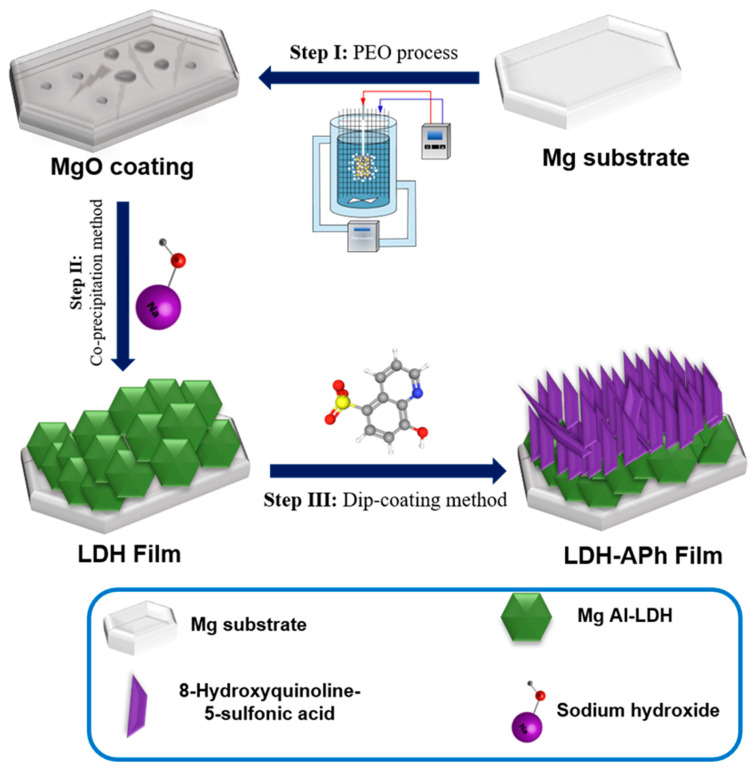
Schematic illustration depicting the synthesis steps of the HQS@LDH-MgO material.

The synthesis of LDH film involved immersing PEO samples in an aqueous solution containing the molecule with chelating properties, sodium nitrate, aluminum and magnesium nitrate. The acquired solutions underwent pH adjustment to reach 10 ± 0.1 through the addition of NaOH solution. Afterward, the hydrothermal step was conducted with electromagnetic stirring at 80 °C for a duration of 8 h. Following the LDH process, LDH-MgO-coated specimens underwent dip chemical coating (DCC) involving the use of the 8-Hydroxyquinoline-5-sulfonic acid compound. Subsequently, the pretreated specimens were submerged in the organic solution for a duration of 12 h to promote the generation of a self-organized organic–inorganic combination. Next, the arranged specimens underwent rinsing with deionized water and ethanol, after which they were allowed to air-dry at ambient room conditions. The resulting procedure is detailed in Figure 1 to elucidate the synthesis steps.

### 2.3. Characterization Methods

The surface characteristics of the prepared samples were examined using field-emission scanning electron microscopy (FE-SEM, Hitachi S-4800, Tokyo, Japan) along with energy-dispersive X-ray spectroscopy (EDS, Horiba EMAX, Tokyo, Japan) for compositional analysis. To analyze constituent compounds and phases and determine the hybrid layer’s surface profile, X-ray diffraction (XRD Rigaku, D MAX-2500, Tokyo, Japan) was employed. Chemical composition analysis was conducted using Fourier transform infrared spectroscopy (FT-IR, PerkinElmer Spectrum 100, Tokyo, Japan) and X-ray photoelectron spectroscopy (XPS, VG Microtech ESCA 2000, PA, USA). Additionally, field-emission transmission electron microscopy (FE-TEM, Tecnai F20, FEI, Tokyo, Japan) at 200 kV was utilized by converting the samples into particles.

### 2.4. Computational Simulations

In this work, the molecule electronic characteristics were calculated using the Gaussian 16 W program [23]. DFT-based simulations were employed to geometrically optimize the molecular structure at the B3LYP/6-311G+ (d, p) molecular level in water by using the Gaussian package’s polarizable continuum model (PCM). After obtaining the optimized geometric configuration of HQS, quantitative analysis on the molecular surface for molecular orbitals and electrostatic potential (ESP) were used to predict reactive sites [24,25]. Using the first principles of DFT implemented in the CASTEP algorithm as part of the Materials Studio package, periodic DFT simulations were carried out to investigate the interfacial mechanism and the adsorption configuration of the organic substance layer on the layered double hydroxides surface [24,25]. The LDH model was constructed using a three-layer slab structure derived from an optimized LDH supercell. HQS@MgAl LDH systems were thoroughly optimized utilizing the Perdew–Burke–Ernzerhof exchange-correlation function (GGA-PBE) within the framework of the generalized gradient approximation [26]. In all computations, electronic wave functions were expanded using a plane-wave cut-off energy of 480 eV. Brillouin zone analysis was performed using a Monkhorst–Pack grid with a smearing parameter of 0.03 Ry. Relaxations were carried out using the Broyden–Fletcher–Goldfarb–Shanno (BFGS) algorithm, with a force convergence cut-off of 0.01 eV/Å per atom [27]. In pursuit of utmost precision in our calculations, we established a rigorous total energy convergence threshold of 10^−5^ eV. To further enhance accuracy, we incorporated the Becke–Jonson damping semiempirical correction scheme, DFT-D3 [28]. The studied surface was created by introducing a 20 Å vacuum along the z-direction. To quantitatively discuss the interfacial mechanism and the mutual interactions of HQS on the LDH surface, the adsorption energy was estimated using the following expression [29]:

(1) $$E_{adsorption}=E_{LDH/HQS}-E_{LDH}{-E}_{HQS}$$

Here, E_LDH/HQS_ (eV), E_HQS_ (eV), and E_LDH_ (eV), respectively, denote the energy of the HQS-Mg-Al LDH composite, the energy of the unadsorbed HQS compound, and the energy of the Mg-Al LDH layer. 

### 2.5. Electrochemical Experiments

An interface 600 potentiostat (Gamry Interface 1000, Philadelphia, PA, USA) was used for potentiodynamic polarization and EIS measurements during the anticorrosion tests in 3.5 weight percent sodium chloride (NaCl) solution. A standard three-electrode cell was employed. The working electrode in this cell consisted of the test specimens with 1 cm^2^ of exposed area, the counter electrode was a Pt plate, and the reference electrode was a Ag/AgCl electrode. Each electrochemical measurement was performed at least three times to ensure repeatability, which was conducted at room temperature. The working electrode was submerged in the solution for 30 min prior to the polarization experiment to obtain a comparatively constant open circuit voltage (OCP). After that, the potential was scanned from −250 mV to +250 mV relative to OCP at a sweep rate of 1 mV s^−1^. The EIS tests were performed in the frequency range from 0.1 to 10^6^ Hz with an amplitude of 10 mV rms sinusoidal perturbation.

## 3. Results and Discussion

### 3.1. Experimental Data

**Figure 2:**
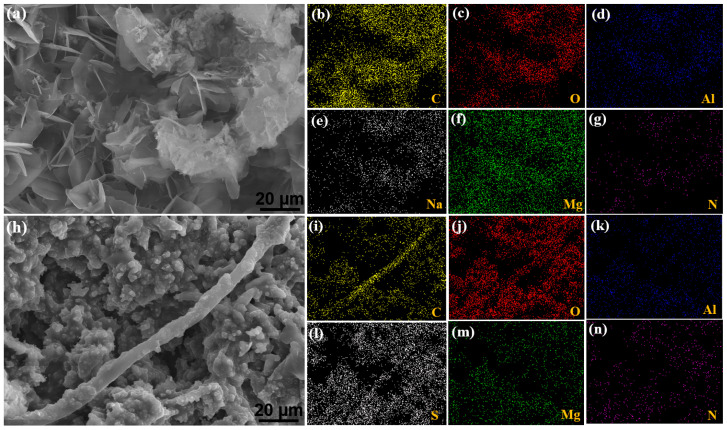
Surface morphologies and the corresponding EDS spectra of (**a**–**g**) the PEO-LDH material and (**h**–**n**) the HQS@LDH-PEO composite.

In Figure 2, microstructures and corresponding EDS results from architectures inspired by nature, including Mg-Al LDH (Figure 2a) and the twig-like HQS@LDH-MgO layer (Figure 2h), are compared. In general, pure MgO exhibits a robust structure characterized by pores of different sizes distributed across a relatively flat film, providing an optimal setting for the initiation, growth, and aggregation of the LDH film. The nano-petals were seamlessly and uniformly incorporated into the PEO surface after in situ formation of the layered double hydroxide as illustrated in Figure 2a. As shown in Figure 2c–g, the EDS mapping demonstrates the presence of Mg, Al, C, and O on the surface of PEO-LDH sample, further supporting the successful loading of the LDH-MgO surface morphology. The duplex structure is formed after the Mg-Al layered double hydroxide has completely precipitated and etched at the MgO surface. In order to create a protective organic film covering the porous layer, the obtained Mg-Al LDH was then subjected to a DCC in HQS solution. During this process, the HQS self-assembly process exhibits an inclination to adhere to the morphological imperfections of the LDH, adopting a twig-like structure. This results in a uniform and comprehensive coverage extending across the entire LDH layer, marked by a high coverage rate and distinctive sealing design features. The resultant micro-twig architecture plays a crucial role in fostering long-term protection for the magnesium alloy. This distinctive configuration positions the final product as a robust contender for providing exceptional protection through the seamless reformation of the surface topography, achieved by the collaborative network structure of HQS molecules with the LDH architecture, as depicted in Figure 2h. The self-assembled material’s element mapping (Figure 2j–n) reveals that all elements are distributed consistently and the distribution of C, S, and O that are higher density than the other elements suggest that the molecules are actively being adsorbed on the surface, further confirming the formation of the organic layer on the coated surface. In addition to offering a substitute for the limitations of autoclaves and permitting a more extensive utilization of LDH systems, the particular morphological alteration resulting from the singular combination of the two bioinspired structures in the presence of the chelating agent has the capability to address intrinsic defects in LDH morphology.

**Figure 3:**
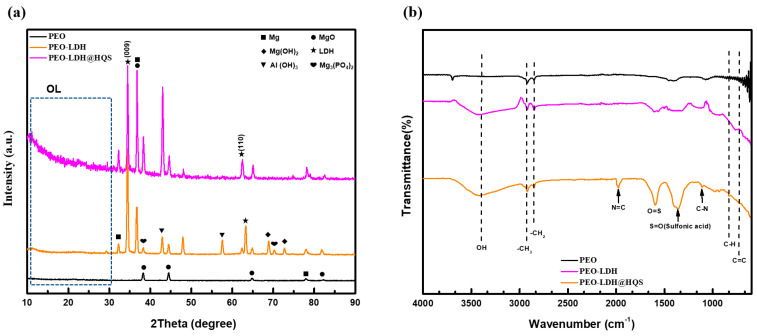
(**a**) XRD patterns and (**b**) FTIR analysis of the treated samples by PEO, treated samples by PEO methods followed by co-precipitation (PEO-LDH), and synthesized HQS@LDH-PEO composite fabricated by PEO and co-precipitation followed by DCC with HQS.

**Figure 4:**
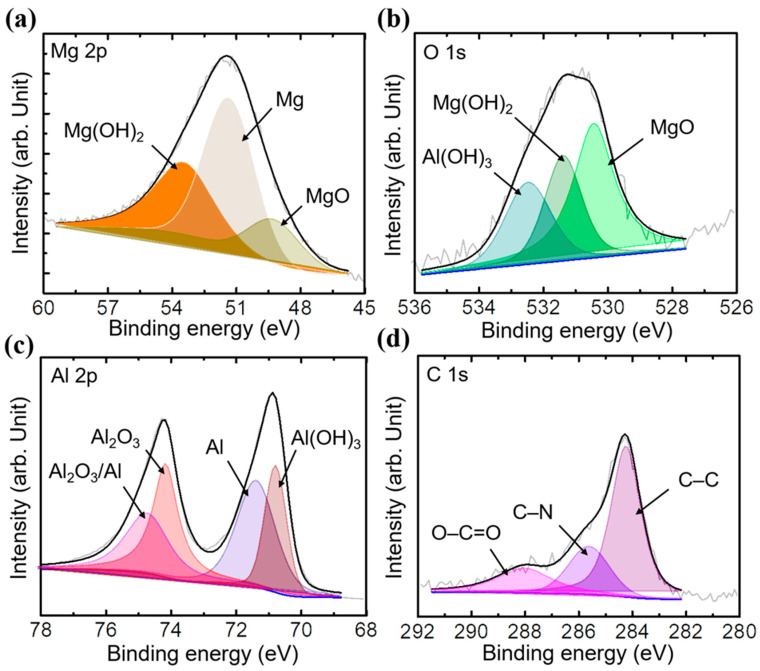
High-resolution XPS profiles of (**a**) Mg 2p, (**b**) O 1s, (**c**) Al 2p, and (**d**) C 1s for the fabricated HQS@LDH-PEO composite.

**Figure 5:**
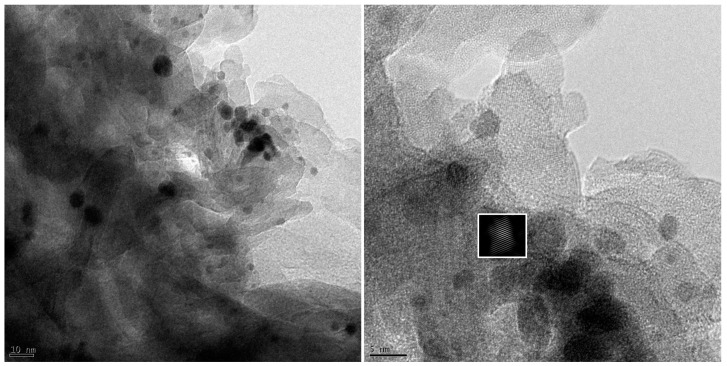
TEM images exhibiting the formation layer-by-layer of the organic molecule on the PEO-LDH material.

By using X-ray diffraction, the crystallization of the inorganic films and the hybrid HQS@LDH-MgO coating created on the metallic surface were further examined. Mg, MgO, and a trace amount of Mg_3_(PO4)_2_ were found in the sample produced by the PEO procedure, as shown in Figure 3a. The peak corresponding to MgO dominates in terms of relative abundance among all peaks, signifying that MgO constitutes the primary oxide within the inorganic layer, according to peak intensities. Additionally, two distinctive XRD peaks at 34.5° and 63.4° were indicative of the pure LDH phases’ (009) and (110) diffraction planes when compared to the pristine PEO, indicating an efficient association between the organic layer and LDH surface. Additional Mg(OH)_2_ and Al(OH)_3_ diffraction peaks were also observed, but with lower intensities, as a result of the interactions between the Mg, Al, and hydroxyl groups. These results show that the LDH layers are successfully applied on the MgO surface. Additionally, the HQS@LDH-MgO particle crystallinity was supported by the XRD patterns. The diffraction peak positions of the hybrid film are identical to the spectrum of the LDH phases, as shown in Figure 3a, proving that the as-fabricated LDH samples remained unaffected following surface modification. The presence of the HQS compound on the LDH layer affected the X-rays’ capacity to adsorb, which somewhat varied the peak intensity, and due to the X-rays considerable depth of penetration, the chemical phase of metallic Mg was identified. As a result of a chemical interaction between the active sites of the organic molecules and the (-OH) pertaining to the LDH framework, additional peaks belonging to the reflection peaks of HQS have instead emerged. In light of this, it is confirmed that the hybrid coating made by fusing the LDH framework and organic layer is verified. Figure 3b displays the FT-IR spectra of the self-assembled material, LDH structure, and PEO coating in order to show the bonding in the studied coatings. As can be seen from Figure 3b, the O-H stretching of hydroxyl groups could be the cause of a distinctive broad band exhibited at 3400 cm^−1^ [30,31]. Similarly, the spectrum of HQS@LDH-MgO affirms the existence of (-OH) groups derived from both the hydroxide groups of LDH and the organic compound, indicative of the deposition of the organic self-assembled layer onto the LDH structure. In the Mg-Al LDH system, the presence of bands at 2920 cm^−1^ and 2840 cm^−1^ can be attributed to the tensile vibration of the C-H group. This observation underscores the role of EDTA as a chelating agent, facilitating the successful production of LDH under ambient pressure [32,33,34,35]. These results are considered as a sign that the chelating molecule effectively forms complexes, demonstrating successful synthesis, and serve as strong confirmation of this conclusion. The appearance of peaks at 1970, 1342, and 1105 cm^−1^, which are attributed to N=C [36,37], S=O [38,39,40,41], and C-N [42,43], respectively, indicates that HQS was adsorbing, which was likely prompted by the emergence of an organic film covering the LDH architecture. Figure 3b provides additional evidence for the existence of the organic self-assembly on the layered coating material. Notably, new characteristic peaks within the range of 960 to 740 cm^−1^ are discerned, indicating the agglomeration of HQS molecules both on the surface and atop the morphological structural defects of LDH architecture. XPS measurements were employed to scrutinize the surface composition and bonding states of constituent elements across diverse coating layers. The hybrid coating’s narrow-scan spectra of Mg 2p, O 1s, C 1s, S 1s, and Al 2p were characterized. As shown in Figure 4a, the high-resolution Mg 2p spectrum may be deconvolved into three sub-peaks identified as MgO, Mg, and Mg(OH)_2_ and located at 49.4, 51.2, and 52.9 eV, respectively [44,45]. After curve fitting, three chemical states of the surface C atom were discovered (Figure 4d); the peak at 283.9 eV corresponds to the energy of the C-C bonds of the aromatic ring in the HQS molecule, and there are two further peaks at 285.9 and 288.1 eV, which are assigned to C-N and C=O bonds, respectively [46]. Additionally, the presence of sulfur highlighted the nucleation and growth behavior of the organic layer, which was manifested as a twig-like structure. Further structural analysis of the produced HQS@LDH-MgO material is performed using transmission electron microscopy, as displayed in Figure 5. The interplanar distances between the organic chains on the lamellar surface are represented by the measured lattice with spacings of 0.37 nm. The outcomes distinctly illustrate the effective interaction among the different components of the duplex structure film, providing strong confirmation of the successful development of the novel material HQS@LDH-MgO.

### 3.2. Computational Perspectives

**Figure 6:**
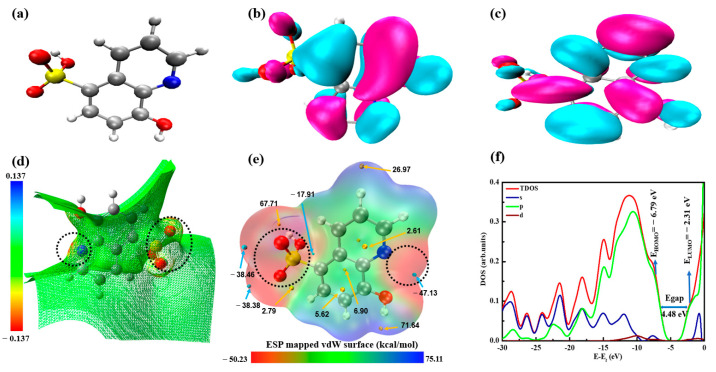
(**a**) Optimized geometry, (**b**) HOMO, and (**c**) LUMO orbitals of HQS molecule. (**d**) ESP statistical distribution on HQS molecular surface, (**e**) ESP-mapped molecular vdW surface with the local surface minima (cyan sphere) and maxima (yellow sphere) points of ESP. (**f**) Energetic position of molecular orbitals with energy gap and the contribution of different atomic orbitals (s, p, d) of HQS on the TDOS.

In order to identify the active sites in the HQS molecule and further clarify the inter- and intra- molecular bonding interactions between HQS and Mg-Al LDH layers, DFT calculations were carried out, as shown in Figure 6. Quantitative analyses of the molecular surface were conducted to obtain the optimized structure and density distribution of the HQS molecule. As shown in Figure 6a,b, the molecular orbital (MO) distribution, i.e., the highest occupied molecular orbital (HOMO) and the lowest unoccupied molecular orbital (LUMO), is localized on various moieties of the HQS molecule, indicating its electron donor–acceptor ability [47]. Figure 6d,e quantitatively describes the electrostatic potential (ESP) distribution and the ESP-mapped van der Waals (vdW) surface for the HQS molecule, and the results show that there is a large portion of molecular surface area with a small ESP value. Among these areas, the negative part mainly corresponds to the contribution of the lone-pair electrons, particularly in the sulfonic acid group and N atom (Figure 6d). In Figure 6e, red, green and blue attribute to ESP distribution varying from −50 to 75 kcal/mol, while the yellow and cyan spheres correspond to the ESP maxima and minima on the vdW surface, respectively. It is clear that the blue area with the ESP minimum points occupies two O atoms in the sulfonic acid group and N atom with ESP values of −38.36, −38.38, and −47.13 kcal/mol [48]. The ESP distribution around the H atom in the O–H group is the global surface maximum, which corresponds to the most positive values (67.71 and 71.64 kcal/mol). Due to the main negative contribution of lone-pair electrons to the electrostatic potential, ESP analysis indicated that the functional groups (SO_3_ and N) can serve as donor and acceptor sites for the intermolecular hydrogen bonding with the hydroxyl groups of the LDH-MgO layers [49]. To reasonably understand the relationship between the electronic properties of HQS and its chemical stability, it is crucial to properly determine the energy difference between HOMO and LUMO [50]. The energy gap offers valuable insights into both the electron donor–acceptor capability and the stability of the HQS molecule. A smaller energy gap, as observed in the empirical data presented in Figure 6f, indicates heightened chemical reactivity or reduced kinetic stability. This is attributed to the lower energy required to excite an electron from the HOMO to the LUMO, thereby promoting chemical interactions [51,52]. With an energy gap of 4.48 eV, the HQS molecule demonstrates a pronounced reactivity facilitating charge transfer and overlap between active centers and the LDH layer, as evidenced by the theoretical findings. Moreover, the molecular orbitals are contributed more by p orbitals, suggesting that the rich π electrons in the HQS molecule promote its electron charge transfer ability [53].

**Figure 7:**
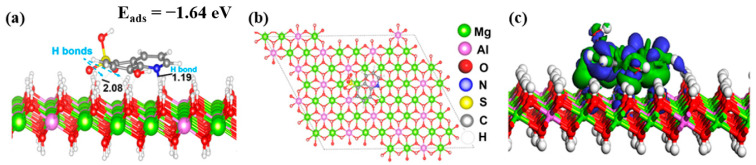
(**a**) Top and (**b**) side views of the parallel adsorption configuration of HQS on the Mg-Al LDH layers indicating the most stable hydrogen-bonded arrangements of HQS and its corresponding adsorption energy as simulated by the DFT. (**c**) The electron density difference distribution for the adsorption of HQS from the side view.

To explore the interfacial mechanism (e.g., adsorption configuration, bonding formation, and electron density difference (EDD)) of the HQS molecule on the Mg-Al LDH surface, first-principles calculations based on DFT were performed, as displayed in Figure 7a–c. It can be seen from Figure 7a that the molecular arrangement of the HQS molecule on the Mg-Al LDH layer is stabilized by the strong intermolecular interactions with hydroxyl groups [54,55]. Calculations reveal that the presence of lone-pair electrons and the π-current of the aromatic ring favor the interaction with the Mg-Al LDH surface. This is confirmed by the bond distances of 1.19 and 2.08 A°, measured from the two O atoms in the SO_3_ group and N atom of the HQS molecule to the surface of the Mg-Al LDH. These results indicate that the hydrogen bond strength is strong. In addition, the adsorption energy (E_ads_) reflects the strong bonding interactions between HQS and Mg-Al LDH layers with −1.64 eV, indicating the vital role of hydrogen bond interaction in the assembly process for the formation of the HQS@Mg-Al LDH composite [10]. Besides, EDD map in Figure 7c shows the accumulation and depletion of electrons at the interface of the Mg-Al LDH hybrid material. The charge distribution at the interface of the HQS@Mg-Al LDH composite is located on the intermolecular bonds formed with hydroxyl groups of Mg-Al LDH, suggesting that the charge transfer takes place between different active regions and Mg-Al LDH layers [56]. Overall, our findings indicate that HQS serves as a suitable linker for generating well-structured self-assemblies on LDH surfaces. This suitability arises from its capacity to act as both a H-bond donor and acceptor, alongside its π-conjugated molecular structure. Additionally, the functional groups play a crucial role in facilitating the inter- and intra- molecular interactions, contributing to the formation of the most stable self-assembled network.

### 3.3. Anticorrosion Property

**Figure 8:**
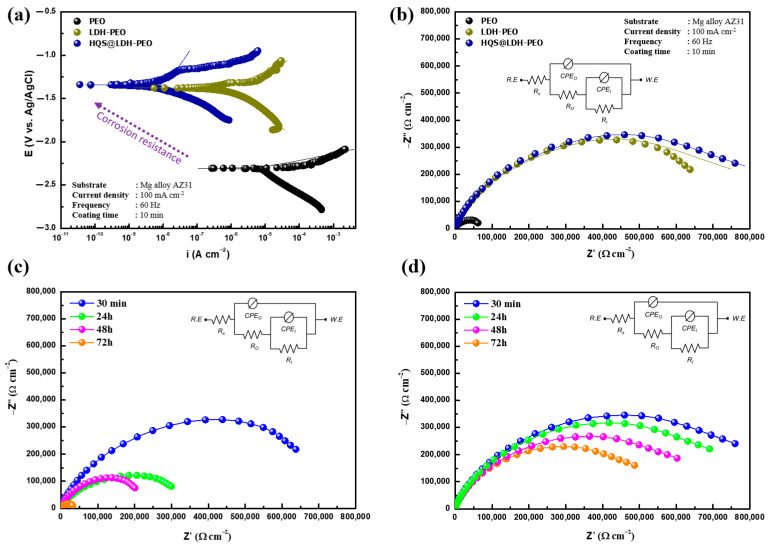
Corrosion properties of different systems after immersion in 3.5 wt.% NaCl solution: short-term (**a**) polarization curves and (**b**) Nyquist plots of PEO, PEO-LDH, and HQS@LDH-PEO composite. Long-term immersion by Nyquist curves of (**c**) PEO-LDH and (**d**) HQS@LDH-PEO.

The nature-inspired structures emerging from both organic and inorganic films have strong anticorrosion performance and also feature an exceptionally durable anticorrosion property for application in a corrosive environment [57]. With a triple electrode system and PDP and EIS measurements, the corrosion-resistant capabilities of the surface films are evaluated in this study. For the MgO, LDH, and HQS@LDH-MgO films, the working electrodes are submerged in a corrosive medium. The electrochemical outcomes of several coatings subjected to the harsh solution for varying immersion durations are shown in Figure 8. Following a short (30 min) immersion in NaCl solution, Figure 8a displays exemplary polarization curves of both the composite coating and uncoated PEO. To measure the rate of immediate corrosion of metals, Tafel polarization is frequently used. In general, improved corrosion resistance is mirrored by a lower corrosion current density (i_corr_) [58,59,60]. The Tafel graphs show that after the creation of LDH and the organic layer, cathodic hydrogen evolution processes as well as cathodic inorganic layer dissolution reactions were all inhibited. Unlike the inorganic layer, the LDH and hybrid material’s polarization curves gradually moved to the upper-left area, where lower current density and greater potential were attained. The results imply that the addition of LDH and HQS layers results in the emergence of new materials, changing their chemical composition and microstructural integrity as well as improving their electrochemical performance, as is evident from the decline in corrosion rate shown in Figure 8a. This result is confirmed by EIS measurement, presented in Figure 8b, which marks a large difference in terms of efficiency between the PEO layer and LDH layer, the latter which inhibits perfectly against the phenomenon of corrosion but only in the short-term. To unravel the diverse electrochemical processes taking place within the organic/inorganic layer, each impedance response was subjected to fitting using an equivalent circuit model that considers the physical and chemical states of the system. In this proposed model, R_S_ represents the solution resistance between the sample and counter electrode, while the outer porous layer and inner barrier layer are denoted by the coating resistance (R_O_) and constant phase element (CPE_O_), respectively. R_I_ signifies the contribution of charge transfer to the overall corrosion resistance, and CPE_I_ represents the double-layer capacitance. Instead of utilizing capacitance to represent the system’s behavior, each resistance is paralleled with CPE. This choice is made because CPEs are better suited for capturing the non-ideal characteristics of the system, stemming from the rough and non-uniform nature of the coating structures. For the long-term, the Nyquist plots of LDH-MgO (Figure 8c) and HQS@LDH-MgO (Figure 8d) are performed for different immersion times. The presented graphs illustrate the temporal evolution of both capacitance and the protective film as each layer is sequentially constructed. Because the LDH porous surface is permeable to the corrosive solution, Nyquist plots for this coating reveal that a notable reduction in the radius of the impedance arc is observed with an extended immersion duration, indicating a decline in its anticorrosion efficacy with time. According to Figure 8d, the duplex structure created by the conjunction of the LDH architecture with the organic film, which can be inferred with the highest capacitance loop and impedance amplitude, significantly improves the anticorrosive capabilities of the Mg substrate over long-term immersion in the aggressive solution, which highlights the stability of our new material. The increases in the Ro values of the LDH-PEO film were notably lower than those observed in the HQS-LDH-PEO film, regardless of the immersion period. This indicates the significance of integrating HQS molecules in stabilizing LDH deposited on the inorganic layer and the underlying Mg alloy substrate in corrosive conditions. The good stability of the HQS@LDH-PEO film was thus clearly corroborated by a higher value of Ro up to 2.10 × 10^6^ Ω cm^2^ after 72 h of exposure to a corrosive solution. Additionally, the reduced CPE_I_ values observed for the HQS@LDH-PEO sample after 72 h of immersion (3.80 × 10^−10^ S·s^n^·cm^−2^) indicate effective suppression of corrosive ion infiltration. This suppression is attributed to the self-assembly of the HQS structure, which covers the entire porous surface of the LDH-PEO coating. This behavior can be linked to the LDH chloride ions’ ability to grab them as well as the HQS’s physical barrier.

## 4. Conclusions

In summary, we have described the fabrication of HQS@LDH-MgO material with nature-mimicking architecture and favorable anticorrosion capabilities as a potent coating for Mg alloy over long-term. Utilizing the inorganic materials as a powerful topic without restrictions, in the improvement of multifunctional nanoparticles, the as-prepared LDH coating has successfully developed in situ with the use of EDTA molecule on the PEO surface under conditions of moderate severity. The present results indicated that LDH layers provided thorough coverage over the PEO coating, effectively sealing its pores and enhancing their functionality. In addition, the research results indicate that by adjusting the arrangement of HQS molecules, hierarchical self-assembly on LDH layers can be achieved, demonstrating remarkable long-term anti-corrosive properties even after 72 h of immersion. Interestingly, the LDH structure and organic self-assembly characteristics would successfully restrict the penetration of chloride ions to the underlying MgO substrate, taking advantage of the anion-exchange potential of the lamellar LDH phases as well as the physical barrier effect of the floret-like architecture. Computational analyses provided insights into the adsorption behavior of HQS molecules and the underlying mechanism of the PEO/LDH hybrid on the Mg substrate, further supporting the validity and significance of the study. The innovative approach suggested in this research not only creates unique structures exhibiting enhanced resistance against corrosion and more practical applications from the perspective of acceptable and suitable experimental settings for various industry sectors, but it also proposes new opportunities for future scientists to build a variety of functional materials.

## Data Availability

Data are contained within the article.
